# Cirrhosis of the Liver: A Case Report and Literature Review of a Rare Case Presentation of Autoimmune Hepatitis With Systemic Sclerosis

**DOI:** 10.7759/cureus.31147

**Published:** 2022-11-06

**Authors:** Shashank Banait, Chetan Burriwar, Jyoti Jain, Priti G Verma, Tanvi Banait, Madhura Joshi

**Affiliations:** 1 Department of Ophthalmology, Jawaharlal Nehru Medical College, Datta Meghe Institute of Medical Sciences, (Deemed to be University), Wardha, IND; 2 Department of Medicine, Mahatma Gandhi Institute of Medical Sciences, Wardha, IND; 3 Department of Obstetrics and Gynaecology, Jawaharlal Nehru Medical College, Datta Meghe Institute of Medical Sciences, (Deemed to be University), Wardha, IND; 4 Department of Medicine, Jawaharlal Nehru Medical College, Datta Meghe Institute of Medical Sciences, (Deemed to be University), Wardha, IND

**Keywords:** systemic sclerosis, cirrhosis of the liver, autoimmune hepatitis, antinuclear antibody, anticentromere antibody

## Abstract

Systemic sclerosis (SSc) is a chronic systemic disease that affects the skin, heart, lungs, kidneys, gastrointestinal tract, and musculoskeletal system. Although gastrointestinal involvement has been reported in approximately 90% of scleroderma patients, liver involvement is uncommon. A 51-year-old female was admitted to the hospital due to abdominal distension and pedal edema. She had a history of Raynaud's syndrome and multiple hypopigmented and hyperpigmented patches over her body for the last year. Her ascetic fluid analysis was transudative with a serum ascites albumin gradient >1.1, and the abdomen and pelvis ultrasonography reported liver cirrhosis with splenomegaly with perisplenic varices. Her antinuclear antibody and anti-centromere antibody were positive. Skin thickening was visible. Her alanine aminotransferase (ALT), aspartate aminotransferase (AST), and serum globulin were raised. Viral serology was negative. We managed her with diuretics, beta-blockers, prednisolone (30 mg/day administered orally), angiotensin-converting enzyme inhibitors, and calcium channel blockers. Edema and abdominal distension decreased with this management, and no Raynaud's phenomenon was observed during the hospital stay.

## Introduction

Systemic sclerosis (SSc) is a rare chronic disorder of unknown etiology, characterized by diffuse fibrosis and generalized vascular abnormalities in the skin, joints, and internal organs, leading to the failure of these organs. The etiology of SSc is multifactorial, with multiple genetic, endogenous, and exogenous factors appearing to interact with disease development. The pathogenesis of fibrosis is complex and is due to the interaction between immunological events and vascular changes, which activate fibrogenic fibroblasts [1]. Autoimmune hepatitis is a chronic inflammation of the liver characterized by the presence of autoantibodies and raised serum globulin levels. It is predominantly a disease that affects women and can present at any age. The disease may begin as acute hepatitis, which may progress to cirrhosis [2]. It is commonly associated with various autoimmune diseases, like autoimmune thyroiditis, type 1 diabetes mellitus, glomerulonephritis, ulcerative colitis, autoimmune hemolytic anemia, and autoimmune thrombocytopenia. However, its association with systemic connective tissue disorders like SSc, systemic lupus erythematosus, and mixed connective tissue disorders is infrequent [3,4]. Here, we report the case of a patient presenting with cirrhosis of the liver due to autoimmune hepatitis along with systemic sclerosis.

## Case presentation

A 51-year-old non-obese female with no history of alcoholism was admitted with complaints of gradually progressive abdominal distension and swelling over both lower limbs for the last month. She had difficulty swallowing 15 days before the presentation, which was gradual and worse with solid food. She had a history of multiple small hypopigmented and hyperpigmented patches around the neck, over the back, forearms, and legs for one year. She also presented with Raynaud's phenomenon, which was characterized by bluish discoloration followed by diffuse pain in her fingers since last year, especially during contact with water. However, there was no history of joint pain or deformity, morning stiffness, fever, pain in the abdomen, diarrhea, constipation, chest pain, or paroxysmal nocturnal dyspnoea or orthopnoea. There was no past history of any neurological deficit like dysarthria, gait abnormalities, dystonia, tremors, diabetes, hypertension, dyslipidemia, metabolic syndrome, rheumatic heart disease, or ischemic heart disease. A family history of liver cirrhosis or any chronic liver disease was obtained to rule out hereditary hemochromatosis, as well as red skin lesions and epistaxis for hereditary hemorrhagic telangiectasia, which were all negative.

**Figure 1:**
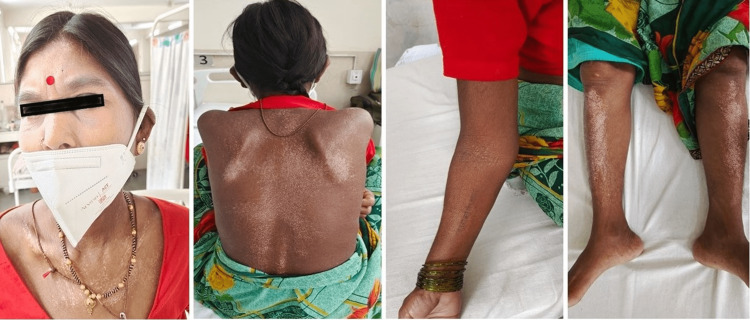
Photographs of the patient show the salt and pepper pigmentation on the skin over the neck, back, forearm, and both legs.

On general examination, she was conscious, afebrile, and pale, with a pulse rate of 100/min and blood pressure of 110/80 mmHg. She was anicteric, and bilateral pitting pedal edema up to the knee was present. Salt and pepper pigmentation around the neck, over the back, forearms, and legs was present (Figure 1).

**Figure 2:**
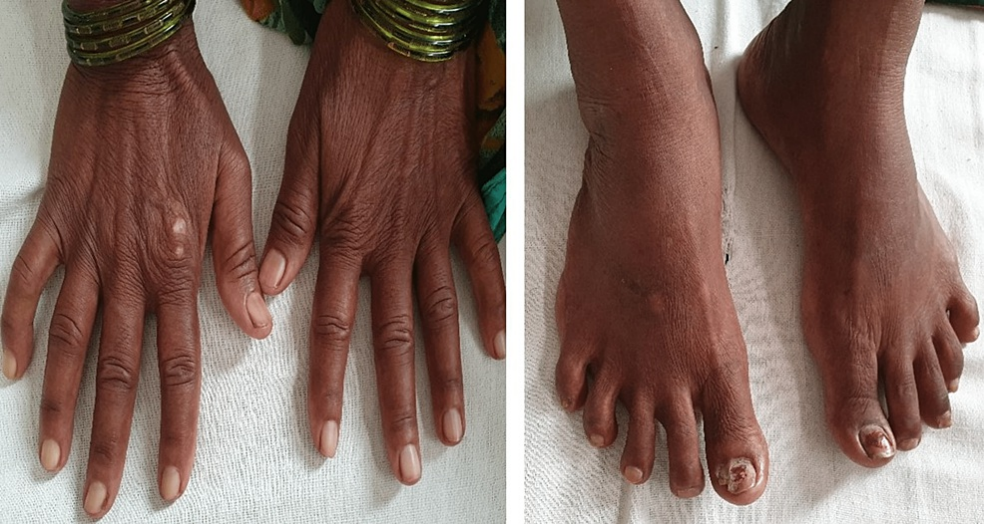
Photographs of the patient show thickening of the skin over the hand and changes in sclerodactyly.

Skin distal to the metacarpophalangeal joint in the hands and at the elbow was tight, but there were no ulcers, swollen fingertips, calcinosis, sclerodactyly, or telangiectasia (Figure 2).

On systemic examination, the abdomen was distended with full flanks, the umbilicus was everted, fluid thrill and shifting dullness were present, and the liver was not palpable, but splenomegaly was present. The rest of the systemic (cardiovascular, respiratory, and central nervous system) examination did not reveal any abnormalities. On slit-lamp examination of the anterior chamber of the eyes, the Kayser-Fleischer (KF) ring was absent. Her hemogram revealed a hemoglobin of 9.6 g/dl, anisopoikilocytosis, microcytic hypochromic red blood corpuscles with a mean corpuscular volume of 71, a white blood corpuscle count of 3.88 with 77.1% granulocytes, and reduced platelets of 0.68 lac/ul. Her liver function tests showed hypoalbuminemia (2.44 gm/dl), hypergammaglobulinemia (4.08 gm/dl), and raised alanine aminotransferase (ALT) of 127 IU/L and aspartate aminotransferase (AST) of 276 IU/L with normal bilirubin and alkaline phosphatase (73 IU/L). Other parameters like blood sugar, serum electrolytes, lipid profile, kidney function tests, and electrocardiogram were normal. Her ascitic fluid was transudative in nature with a serum ascites albumin gradient of 1.35. Her blood, urine, and ascitic fluid cultures did not show the growth of any bacteria. The presence of bilateral pitting pedal edema, ascites, splenomegaly, hypoalbuminemia, and a serum ascites albumin gradient of 1.35 led to the diagnosis of liver cirrhosis with portal hypertension [5,6].

**Figure 3:**
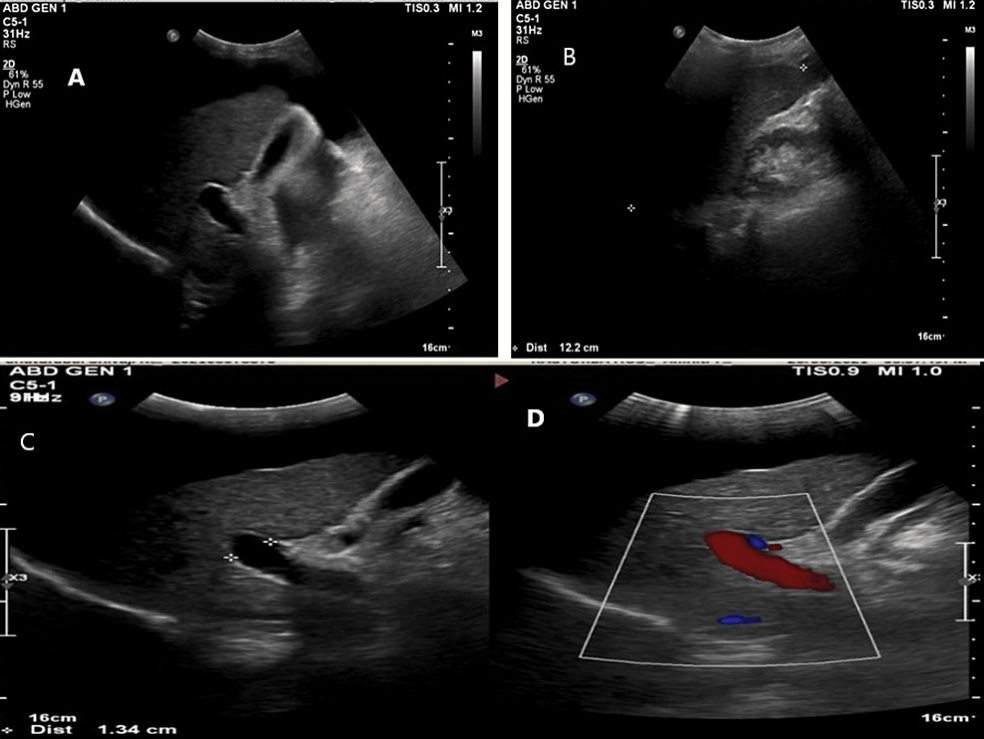
Ultrasonography of the abdomen and pelvis of the patient showed (A) a normal gallbladder with no dilatation; (B) evidence of a small-sized (9.7 cm), shrunken liver with coarsened echotexture of parenchyma and irregular margins with a normal biliary tree; (C) portal vein dilation (12 mm); (D) normal blood flow in the portal vein on color doppler imaging.

Ultrasonography of the abdomen showed a small-sized (9.7 cm) shrunken liver with coarsened echotexture of parenchyma with irregular margins, suggestive of liver cirrhosis with gross ascites, periportal fibrosis, and splenomegaly (14.1 cm), a dilated portal vein of 12 mm with the normal color flow on Doppler imaging, a dilated splenic vein of 15 mm, and a partially distended gall bladder. The common bile duct and intra-hepatic biliary radicals were normal (Figure 3).

**Figure 4:**
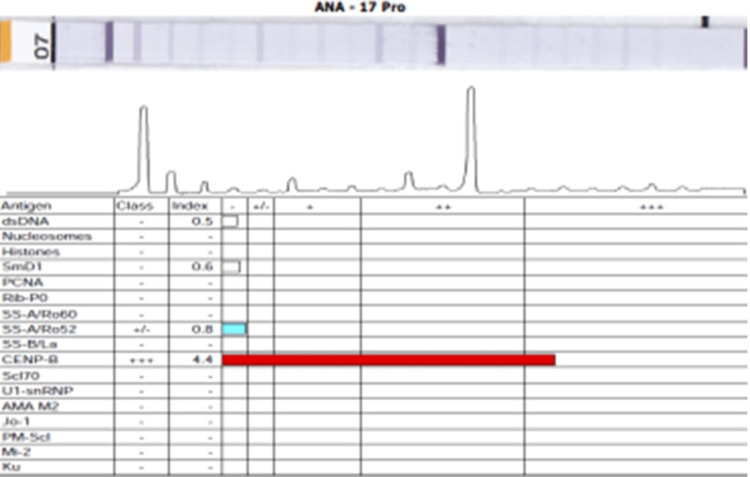
The report of the immunological profile of the patient.

Her fibroscan of the liver could not be done due to the unavailability of this facility at our rural hospital. Her viral serology, hepatitis B surface antigen (HBsAg) for hepatitis B, antibody to hepatitis C virus (anti-HCV), and quantitative hepatitis C virus ribonucleic acid (HCV RNA) by polymerase chain reaction (PCR) and human immunodeficiency virus (HIV) infection test by enzyme-linked immunosorbent assay (ELISA) method were negative. Her serum ferritin of 69 ng/ml, serum ceruloplasmin of 30 mg/dL, and 24-hour urinary copper of 20 µg/day were normal. Her anti-mitochondrial antibodies were negative. Antinuclear antibody 17 profile blot (ANA17B) and anti-centromere antibody with a class index of four were positive (Figure 4).

On the American College of Rheumatology (ACR)/European Alliance of Associations for Rheumatology (EULAR) criteria for the classification of SSc (2013), her total score was 15. (nine for skin thickening and three each for Raynaud's phenomenon and positive anti-centromere antibody [7]. A liver biopsy could not be performed as the patient did not give consent.

The provisional diagnosis of SSc with autoimmune hepatitis leading to cirrhosis of the liver was considered as per the international autoimmune hepatitis group diagnostic criteria [2] (Table 1).

**Table 1:** Diagnostic criteria for autoimmune hepatitis (international autoimmune hepatitis group). ALT: alanine aminotransferase; ANA: antinuclear antibody; Anti - LKM - 1: Anti – Liver kidney microsomal – 1 antibody; ASMA: anti-smooth muscle antibody; AST: aspartate aminotransferase.

Diagnostic criteria	Patient values
1. A minimum of one elevated ALT or AST > twice the upper limit of the normal reference range	Her ALT and AST were raised > two times the upper limit of the reference range.
2. A minimum of one positive laboratory test (elevated gamma globulin or positive ANA/ASMA) or anti-LKM-1	She had hypergammaglobulinemia and a positive ANA. We were unable to conduct ASMA and anti-LKM-1 antibody assays due to financial constraints.
3. Exclusion of other cirrhosis etiologies	We excluded chronic viral hepatitis (hepatitis B and C), alcoholic liver disease, hemochromatosis, non-alcoholic fatty liver disease, primary sclerosing cholangitis, biliary cirrhosis, right-sided heart failure, medications (e.g., methotrexate and isoniazid), Wilson's disease, alpha-1 antitrypsin deficiency, celiac disease, polycystic liver disease, hereditary hemorrhagic telangiectasia, idiopathic adulthood ductopenia, granulomatous liver disease, infection (e.g., brucellosis, and echinococcosis) on the basis of history, examination and or biochemical, immunological, and radiological investigations.

She was managed with diuretics, beta-blockers, angiotensin-converting enzyme inhibitors, calcium channel blockers, and steroids. She showed significant improvement in her symptoms; her edema and abdominal distension decreased, and no Raynaud's phenomenon was observed during the hospital stay.

## Discussion

Systemic sclerosis is a multisystem disorder that primarily affects the skin, heart, lungs, gastrointestinal tract, musculoskeletal system, and kidneys. The disease is characterized by tissue fibrosis, vasculopathy, and an autoimmune response associated with specific autoantibodies. Gastrointestinal involvement is common, up to 95% [8]. However, the liver is rarely involved in this disease. As shown in the review, eight (1.1%) of 727 patients with scleroderma had involvement of the liver [9]. Primary biliary cirrhosis is the most commonly found hepatic disease in patients with SSc, while autoimmune hepatitis is rare [10,11,12].

Although the pathogenesis of the development of autoimmune hepatitis in patients with SSc is still unclear, it may be due to a dysregulated response of the cellular and humoral immune systems. According to the literature, anti-centromere antibodies are found in 3-13% of patients with autoimmune hepatitis [1,9]. Our patient was diagnosed with autoimmune hepatitis according to the criteria proposed by the international autoimmune hepatitis group, and she fulfilled all the diagnostic criteria as mentioned in Table 1 [2,13].

A few cases of autoimmune hepatitis without primary biliary cirrhosis in SSc patients have been reported. In three cases, SSc was diagnosed before the development of autoimmune hepatitis, while in one case, both conditions were diagnosed simultaneously [14-17]. The clinical characteristics of five patients and their laboratory profiles, along with the patients described in the current report, are summarized in Table 2.

**Table 2:** Clinical characteristics of autoimmune hepatitis cases with systemic sclerosis described in English literature and chosen for this study. ALT: alanine aminotransferase; anti-centromere: anti-centromere antibody; AST: aspartate aminotransferase; CREST: calcinosis, Raynaud’s phenomenon, oesophageal dysmotility, sclerodactyly, and telangiectasia syndrome; CRST: calcinosis cutis, Raynaud’s phenomenon, sclerodactyly, telangiectasia syndrome; FANA: fluorescent anti-nuclear antibody; g/dL: grams/deciliter; g/L: Grams/liter; IgG: immunoglobulin G; IU/L: international units /liter; lacs/ul: lacs/microliter; mg/day: milligrams/day; mg/dl: milligrams/deciliter; SSc: systemic sclerosis.

Author [Reference]	Present case	You et al [14]	Marie et al. [15]	Marie et al. [15]	Ishikawa et al. [16]	Yabe et al. [17]
Year of publication	-	2012	2001	2001	1995	1992
Country	India	Korea	France	France	Japan	Japan
Age/Sex	51/ Female	51/ Female	67/ Female	48/ Female	48/ Female	51/ Female
Presenting symptoms	Abdominal distension, swelling over both lower limbs, Raynaud’s phenomena, dysphagia	Hematemesis, Raynaud’s phenomena	Raynaud’s phenomena, arthralgia, pain in the right hypochondrium	Raynaud’s phenomena, dysphagia	Raynaud’s phenomena, arthralgia	Raynaud’s phenomena, fatigue, digital ulcer
Clinical findings	Diffuse SSc, pedal edema, ascites, splenomegaly	Limited SSc	CREST syndrome	CRST syndrome	CREST syndrome	CREST syndrome, Jaundice
Lab investigations	Hemoglobin-9.5 g/dL, platelets - 0.68 lacs/uL, Serum albumin-2.44 g/dL, serum globulin-4.08 g/dL, total bilirubin-ALT-127 IU/L AST-276 IU/L	Hemoglobin - 8.2 g/dL, platelets – 0.74 lacs/uL, serum albumin -2.44 g/dL, IgG -16.17 g/l, total bilirubin- 1.4 mg/dL AST-124 IU/L ALT-54 IU/L	Serum albumin-3.8 gm/dL, IgG-21 g/l, total bilirubin-5 mg/dL ALT-240 IU/L, AST-223 IU/L	Serum albumin-3.8 g/dL, IgG-35 g/L, total bilirubin-5 mg/dL ALT-175 IU/L, AST-147 IU/L	Hemoglobin-13.3 g/dL, WBC count-3800/uL, total bilirubin-0.6 mg/dL, serum globulin-2.8 g/dL, ALT-260 IU/L, AST-214 IU/L	Hemoglobin-12.4 g/dL, WBC count-7800/uL, IgG-20.05 g/L, total bilirubin-29.1 mg/dL, ALT-295 IU/L, AST-453 IU/L
Autoantibody	Antinuclear antibody 17 profile blot, Anti-centromere antibody (+)	FANA 1280× Anti-centromere (+)	FANA 600× Anti-centromere (+)	FANA 1000× Anti-centromere (+)	FANA 2560× Anti-centromere (+)	FANA 640× Anti-centromere (+)
Treatment	Prednisolone 30 mg/day	Prednisolone 30 mg/day with azathioprine	Prednisolone 40 mg/day	Prednisolone 35 mg/day and azathioprine	Not mentioned	Prednisolone 40 mg/day
Outcome	Improvement in clinical symptoms and liver function test	Improvement of liver test abnormalities	Marked improvement of liver test abnormalities	Completely normal liver function test	Not mentioned	Complete disappearance of liver test abnormalities

In our case, cirrhosis of the liver due to autoimmune hepatitis and SSc was diagnosed during the current admission. However, her fibroscan of the liver could not be done due to the unavailability of this facility at our rural hospital, and a liver biopsy could not be performed as the patient did not give consent. In the present case, liver involvement was advanced compared to the previous four cases. These previous case reports indicate that patients with SSc are at an increased risk of developing autoimmune hepatitis (Table 2).

This case was diffuse SSc in which the patient had Raynaud's phenomenon, skin thickening in the hands, feet, and elbows, and salt and pepper pigmentation skin over the back and trunk also, but there were no calcinosis cutis, sclerodactyly, or telangiectasia. High-dose oral corticosteroids and azathioprine are the drugs of choice for treatment as they effectively alleviate symptoms and the outcome of autoimmune hepatitis [18].

Managing autoimmune hepatitis is difficult with diffuse cutaneous SSc patients, as these patients may develop a renal crisis with high-dose steroid therapy [19,20]. Future studies are required to determine the treatment outcome and complications of high-dose steroid therapy for combined autoimmune hepatitis and SSc.

## Conclusions

Patients with SSc and autoimmune hepatitis should be observed for the development of other autoimmune diseases. A high degree of observation is necessary to diagnose autoimmune hepatitis in patients with SSc as symptoms are non-specific and may appear gradually over years. Patients with SSc should be monitored for liver function tests regularly as early diagnosis and treatment of autoimmune hepatitis will help achieve a reasonable remission rate.
